# Prediction of Slot Shape and Slot Size for Improving the Performance of Microstrip Antennas Using Knowledge-Based Neural Networks

**DOI:** 10.1155/2014/957469

**Published:** 2014-10-28

**Authors:** Taimoor Khan, Asok De

**Affiliations:** Department of Electronics and Communication Engineering, National Institute of Technology, Patna 800005, India

## Abstract

In the last decade, artificial neural networks have become very popular techniques for computing different performance parameters of microstrip antennas. The proposed work illustrates a knowledge-based neural networks model for predicting the appropriate shape and accurate size of the slot introduced on the radiating patch for achieving desired level of resonance, gain, directivity, antenna efficiency, and radiation efficiency for dual-frequency operation. By incorporating prior knowledge in neural model, the number of required training patterns is drastically reduced. Further, the neural model incorporated with prior knowledge can be used for predicting response in extrapolation region beyond the training patterns region. For validation, a prototype is also fabricated and its performance parameters are measured. A very good agreement is attained between measured, simulated, and predicted results.

## 1. Introduction

Modern wireless communication systems like satellite communication, radar communication, global positioning satellite (GPS) system, and so forth are demanding more accurate and efficient modeling schemes for microstrip antennas (MSAs). Because of their operation in dual-frequency mode, the MSAs have eliminated two single-frequency operated antennas in these applications. Although the microstrip patch antennas have many inherent attractive features like low profile, conformable to planar and nonplanar surfaces, low fabrication cost, and so forth, they still suffer from the drawbacks of poor radiation characteristics (narrow bandwidth, low gain, low efficiency, etc.) which require more attention [1]. To overcome these drawbacks, slot is inserted on the radiating surface of the patch antennas. To achieve the desired level of performance parameters (resonance frequency, gain, directivity, antenna efficiency, radiation efficiency, etc.), actual shape and appropriate size of the introduced slot needs to be determined. Creating a flexible analytical model for this purpose is still a challenging task in electromagnetic community [2]. Electromagnetic simulation like method of moment (MoM) based IE3D software [3] can do it but only at the cost of large computational time. The simulation approach is not suitable in the situation where instant answer is required as in the case of synthesizing the microstrip antennas by the antenna designers. To reduce the computational time, neural networks modeling is preferred which predicts the response very fast after being trained properly. The artificial neural network (ANN) models provide a common outline for modeling complex geometries and are much faster than the simulation approaches and more accurate than the polynomial fitted methods and the empirical models [2]. These models allow more input dimensions than look-up table methods and are easier to develop when a new geometry is introduced. In last decade, ANN models have received much attention in microwave community due to their ability and adaptability to learn from experience during training and generalize from previous examples to new ones, fast real-time operation, and ease of implementation features [2]. The trained neural model predicts the response to be approximately equal to its measured or simulated counterpart very fast for every small change in the applied input pattern. Different neural models have been proposed for modeling the microstrip patch antennas [4–12], slotted microstrip antennas [13, 16–21], and the reflectarray antennas [14, 15]. The neural models [4–21] may not be very reliable without having adequate number of training patterns. In addition, even with sufficient training patterns, the reliability of neural model when used for extrapolation purpose is not guaranteed and in most cases is very poor. The learning patterns for the neural models are generally created using simulation and/or measurement approach. For a complex geometry, generating large amount of training patterns becomes time consuming and sometimes very expensive because simulation/measurement approach is to be performed for several combinations of each input parameter associated to that geometry.

Towell and Shavlik [22] have firstly proposed a concept of reducing the required training patterns by incorporating prior knowledge in the standard neural models. The incorporated knowledge provides additional information about the original problem which may not be adequately represented in the limited training patterns. Wang and Zhang [23, 24] have embedded prior knowledge into internal neural networks structures in the form of empirical functions to resolve three different microwave design problems. Watson et al. [25, 26] have proposed knowledge-based neural networks (KBNN) model for microwave components modeling. Dandurand and Lowther [27] have used KBNN model for identifying the performance of electromagnetic devices. Watson et al. [28] have designed wideband coplanar waveguide patch/slot antennas using KBNN model. Wang et al. [29] have modeled stripline discontinuities by neural networks with knowledge-based neurons. Zingg and Gupta [30] have designed microwave reflection and loaded types phase shifters using knowledge-aided-design (KAD) neural networks. Hong and Wang [31] have used neural networks with knowledge-based neurons in hidden layer for modeling microstrip T-junction. Devabhaktuni et al. [32] have, recently, proposed an efficient knowledge-based automatic model generation (KAMG) technique for microwave modeling. Some innovative strategies including knowledge-based microwave design and optimization have been proposed by Rayas-Sánchez [33]. Rayas-Sanchez and Zhang [34] have proposed KBNN based advanced electromagnetic data sampling algorithms for modeling several microwave structures. Devabhaktuni et al. [35] have introduced a novel ANN-based reverse-modeling approach for efficient electromagnetic compatibility (EMC) analysis of printed circuit boards (PCBs) and shielding enclosures. In the literature [4–34], neither a standard nor a knowledge-based neural model has been proposed for predicting the shape and size of a slot introduced on the radiating surface of the microstrip patch antenna. It is very essential for the antenna designers to instantly predict the actual shape and appropriate size of the introduced slot for achieving the desired level of performance parameters. In the proposed work, a standard neural networks modeling is firstly discussed for predicting the shape and size of the introduced slot on the radiating surface of a rectangular patch microstrip antenna. A knowledge-based neural network model is then created for reducing the number of training patterns without deteriorating the computed accuracy. A prototype of microstrip antenna is also fabricated and analyzed using network analyzer. A very good agreement is achieved between the results of KBNN model, measured results, and simulated results which support the effectiveness of the proposed work.

## 2. Geometry for Patterns Generation

**Figure 1:**
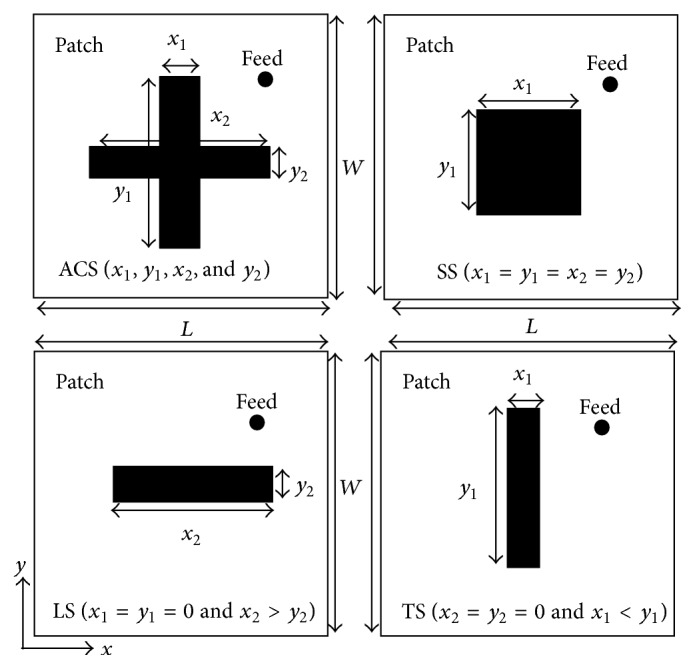
Proposed microstrip antenna with different slots.

The cross-sectional view of the proposed microstrip patch antenna with four different slots is shown in Figure 1. A rectangular patch antenna of size 61 × 56 mm^2^ is designed using RT-Duroid substrate RO3003 (*ε*_*r*_ = 3,* h* = 0.762 mm, and tan*δ* = 0.0045). Two resonating modes (TM_10_ and TM_01_) are excited by a single probe for getting dual resonance. The performance of the patch antenna is further improved by inserting air gap between the substrate sheet and the ground plane [40]. To make the analysis simpler, it has been decided to consider four cases: asymmetrical cross slot (ACS), square slot (SS), longitudinal slot (LS), and transverse slot (TS), respectively. These four geometries are analyzed using method of moment based IE3D software [3] on a personal computer with system configuration, Dell Optiplex 780 Core 2 Duo CPU E8400, 3.0 GHz with 4.0 GB RAM. Different patterns for dual resonance, dual-frequency gains, dual-frequency directivities, dual-frequency antenna efficiencies, and dual-frequency radiation efficiencies are generated by varying the slot dimensions denoted by* x*_1_,* y*_1_,* x*_2_, and* y*_2_ for ACS-, SS-, LS-, and TS-slotted geometries, respectively. Thus, ten different electrical parameters are achieved for each set of four geometrical parameters. Total of 1960 patterns (490 for each case) are generated by varying slot dimensions as follows: 1 mm ≤ slot dimensions ≤ 50 mm in IE3D software [3]. For generating these patterns, a sampling step of 0.1 mm is used during the sampling of the slot dimensions. The 1960-simulated patterns used for training and testing of the standard neural networks model are to be discussed in Section 3.

## 3. Standard Neural Networks Modeling

**Figure 2:**
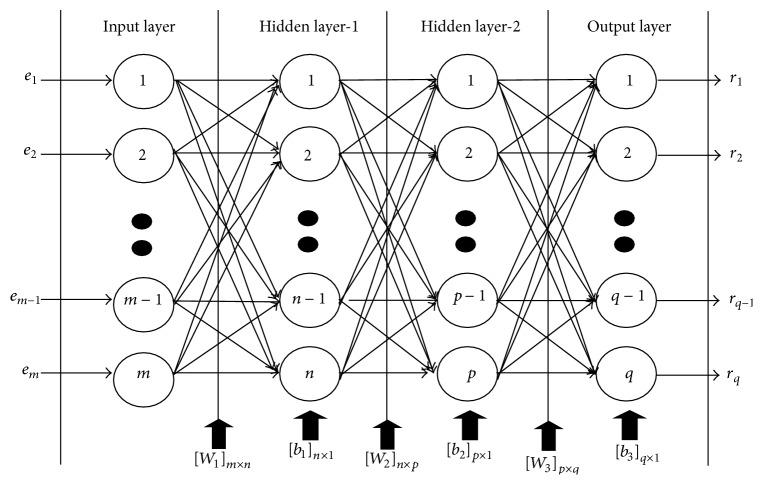
MLP neural networks with two hidden layers.

Artificial neural networks (ANNs) are extremely distributed analogous processors and becoming powerful techniques for resolving the problems which are cross disciplinary in nature. Multilayered perceptron (MLP) neural networks consist of an input layer, a number of hidden layers, and an output layer in which each layer is having entirely different role. The neural networks have usual tendency for storing the empirical knowledge during training and making it available for use during testing. The aim of training process is to minimize the error between actual output and calculated output from the neural model. In general, three common steps are used to train the MLP neural networks. Firstly, the training patterns are generated, the structural configuration of hidden layer is then optimized in the second step, and, finally, the weights and biases are then optimized in the third step using training algorithm. The trained neural model, thus obtained, is tested on some arbitrary sets of patterns which are not used during training of the model. Training and testing algorithms for the neural networks model are implemented in MATLAB software [41] on a personal computing machine. In fact, the neural model produces results very fast after being trained on the training patterns but the generation of patterns and allocating them into training and testing patterns are challenging tasks for a complex geometry of microstrip antennas. An MLP neural networks model with two hidden layers is shown in Figure 2 in which the structural configuration of the distributed neurons is mentioned as *m*∗*n*∗*p*∗*q* where* m*-,* n*-,* p*-, and* q*- represent the number of neurons in the input layer, first hidden layer, second hidden layer, and in output layer, respectively. Further, [*W*_1_], [*W*_2_], and [*W*_3_] represent the weight matrices between input layer to hidden layer-1, hidden layer-1 to hidden layer-2, and hidden layer-2 to output layer, respectively. The bias value at hidden node-1, hidden node-2, and output node is denoted by [*b*_1_], [*b*_2_], and [*b*_3_], respectively. Initially, some random numbers are assigned to the weights and biases corresponding to an applied input pattern. Adjustment in the weights and biases is carried out during training of the model using training algorithm to get a desired value of response corresponding to the applied input pattern. The training performance of the model is observed for seven different algorithms: BFGS quasi-Newton (BFG), Bayesian regulation (BR), scaled conjugate gradient (SCG), Powell-Beale conjugate gradient (CGB), conjugate gradient with Fletcher-Peeves (CGF), one-step secant (OSS), and Levenberg-Marquardt (LM), respectively [36–39]. During pattern generation, it is observed that the uniform variation in geometrical parameters produces nonuniform variation in electrical parameters which causes convergence problem during training of the neural model. The problem is resolved by normalizing both the geometrical and the electrical parameters between +0.1 and +0.9 in MATLAB software before applying training.

The standard MLP neural model is proposed to predict the actual shape and appropriate size of the introduced slot for achieving the desired level of dual resonance (*f*_1_ and* f*_2_), dual-frequency gains (*G*_1_ and* G*_2_), dual-frequency directivities (*D*_1_ and* D*_2_), dual-frequency antenna efficiencies (*A*_1_ and* A*_2_), and dual-frequency radiation efficiencies (*R*_1_ and* R*_2_). There are four shapes to be predicted in the proposed work: asymmetrical cross slot (ACS), square slot (SS), longitudinal slot (LS), and transverse slot (TS). The predicted shape is being represented here by a dummy variable “*S*” where* S* = 1, 2, 3, and 4 corresponds to ACS, SS, LS, and TS, respectively. The predicted size of the introduced slot is represented by dimensions* x*_1_,* y*_1_,* x*_2_, and* y*_2_. Thus the response matrix is designated as [*R*] → [*x*_1_ *y*_1_ *x*_2_ *y*_2_ *S*] for the excitation matrix [*E*]→[*f*_1_ *f*_2_ *G*_1_ *G*_2_ *D*_1_ *D*_2_ *A*_1_ *A*_2_ *R*_1_ *R*_2_]. Using trial and error method, the structural configuration shown in Figure 2 is optimized as* m* = 10,  *n* = 73, * p* = 76, and* q* = 5 for the best performance. Here 10 neurons in the input layer are used for applying the excitation and 5 neurons in the output layer are used for getting the response. The MLP neural model is trained by considering some initial parameters: mean square error (MSE) = 4.79 × 10^−5^, learning rate (*η*) = 0.15, and momentum coefficient (*μ*) = 0.004. The trained model then predicts the appropriate shape and size of the introduced slot very fast for any arbitrary set of resonance frequencies (1.5 GHz ≤ resonance frequencies ≤ 3.0 GHz), gains (6.2 dBi ≤ gains ≤ 9.6 dBi), directivities (6.6 dBi ≤ directivities ≤ 9.9 dBi), antenna efficiencies (83% ≤ antenna efficiencies ≤ 100%), and radiation efficiencies (85% ≤ radiation efficiencies ≤ 100%) for dual-frequency operation.

## 4. Knowledge-Based Neural Modeling

**Figure 3:**
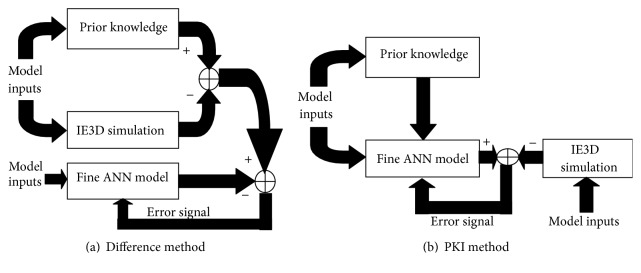
Knowledge-based ANN modeling [25, 26].

The standard MLP neural networks model discussed in Section 3 is trained with 1372 patterns (~70% of total generated patterns) and tested for the rest 588 patterns. To generate such a large number of training patterns is a time consuming process and sometimes it becomes very expensive. It is so because the simulation/measurement approach is to be performed for several combinations of each input parameter associated to that geometry. Without degrading the performance of the neural model discussed in Section 3, two methods, difference method and prior knowledge input (PKI) method, are used for reducing the required number of training patterns as shown in Figure 3.

In the difference method, the fine ANN model is trained on the difference between the simulated outputs and the outputs of coarse ANN model. In PKI method, on the other hand, the outputs of coarse model are used as inputs for fine ANN model in addition to simulated inputs. Thus, the input/output mapping of the ANN model is in between the output response of the existing model and that of the target model. In the proposed work, the prior knowledge is attained by an already trained neural model, known as coarse model. It can also be attained by a set of analytical equations or empirical models. This coarse model contains some information about the behavior of the proposed problem and does not produce the desired level of accuracy. But when it is incorporated in the two methods shown in Figure 3, the overall accuracy increases drastically and the required training patterns significantly reduces. The optimization strategy for structural configuration and the training of the coarse and fine ANN models is similar to the standard MLP neural model discussed in Section 3. In the difference and PKI methods, the structural configuration of coarse model and fine models are optimized as discussed in MLP neural modeling discussed in Section 3 and the optimized values are mentioned in Table 1.

## 5. Computed Results and Validation

A standard MLP neural modeling scheme is proposed for instantly predicting the actual shape and appropriate size of the inserted slot on rectangular radiating surface of microstrip antenna for achieving desired level of performance parameters. The training performance of the proposed standard neural model is observed for seven training algorithms: BFG, BR, SCG, CGB, CGF, OSS, and LM [36–39]. But, for the proposed problem, only Levenberg-Marquardt (LM) back propagation algorithm is proved to be the most accurate training algorithm as mentioned in Table 2. Further, it is also proved to be the fastest converging training algorithm as it requires the least training time (1589 sec.) with the least number of iterations (i.e., 14562).

Table 3 shows a comparison of model size, number of weights, and bias values used in three neural models. The numbers of weights and biases for MLP model are computed as 6658  (10 × 73 + 73 × 76 + 76 × 5) and 154  (73 + 76 + 5), respectively. Similarly for the difference method, these are computed as 400  (10 × 8 + 8 × 5 + 15 × 14 + 14 × 5) and 32  (8 + 5 + 14 + 5), respectively. In the same way for PKI method, the weights and biases are computed as 500 and 34, respectively. Thus the difference and PKI methods require only 6.01% and 7.51% of the weights required in standard MLP neural model, respectively. Similarly, the numbers of bias values in these two methods are only 20.78% and 22.08% of the bias values required in MLP neural model, respectively. It is concluded here that, by using KBNN methods, the number of weights are reduced by 93.99% and 92.49% and biases by 79.22% and 77.92% in the difference method and PKI method, respectively, at the cost of slight deterioration in the computed accuracy.

The accuracy comparison in three models is given in Table 4. The average testing error in standard MLP neural model is drastically increasing by reducing the training patterns from 70% to 40%. It means that the MLP neural model produces more accurate results if it is trained with adequate number of training patterns. On the other hand, two knowledge-based neural methods are producing more accurate results even for lesser training patterns. The testing accuracy of the difference method is deteriorating from 0.57% to 1.53% by reducing the number of training patterns from 70% to 40%, whereas, in PKI method, it is only from 1.15% to 2.23% for the same level of reduction in the training patterns. Thus, the difference method is observed to be more accurate than the PKI method for the proposed problem.  Table 5 depicts a comparison between simulated and predicted slot sizes along with their simulated dual resonances which shows a very good agreement between the two.

As the weights and biases in the proposed neural modeling schemes are selected randomly for initialization, the computed errors in these three neural models have also been analyzed by considering the stochastic behavior of mean and standard deviation of the computed errors [42]. A relationship between mean and standard deviation of an error set is developed by considering the coefficient of variation (CoV) which is defined as the ratio of standard deviation to the mean value. CoV closer to 0, represents greater uniformity of error, whereas CoV closer to 1, represents the larger variability of the error. For difference method, the mean in four-dimensional errors is computed as 1.4543, 1.3487, 1.4673, and 1.5143, respectively, whereas the standard deviation is computed as 0.1499, 0.1314, 0.1566, and 0.1549, respectively. Hence, the coefficient of variations for these four values is coming out to be 0.1031, 0.0974, 0.1067, and 0.1023, respectively, which shows that all the error points over a full validation set of 1960-simulated patterns are uniformly distributed [15].

During simulating a structure in IE3D software, from 1.5 GHz to 3.0 GHz frequency range with total 100 sampling points is used. For the proposed geometry, the simulation time is computed as ~1 h 53 min. per structure and 36 MB system memory (RAM) is required for each simulation. By using neural networks modeling, the computational time and the required memory storage are fairly reduced. The time elapsed during training of the MLP neural model is computed as 1589 sec. whereas ~49 msec. time is elapsed in producing the results after training [42]. Thus, it is concluded here that the neural model after training is much faster than that of the conventional electromagnetic simulators. Further, the training of the model requires only 29 KB RAM of a system and, for testing the performance, only 1.33 KB RAM is required. Hence, the required memory space in training as well as in testing of the neural networks model is also lesser as comparison to 36 MB required for simulation.

**Figure 4:**
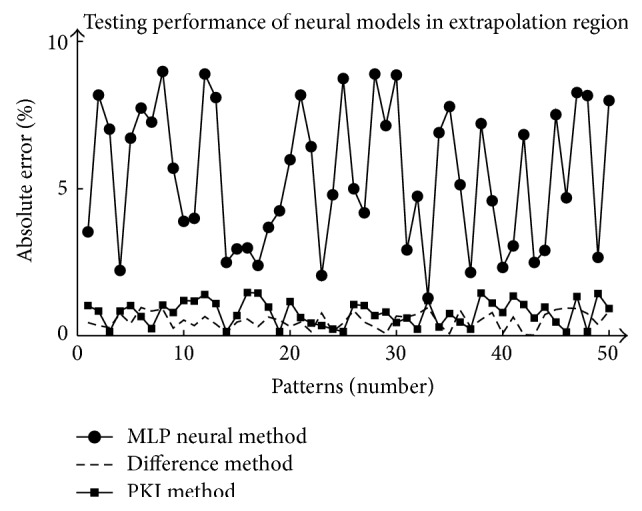
Performance of ANN models in extrapolation region.

The proposed neural modeling schemes are also tested in extrapolation region (outside the region of generated training patterns) of the input space which is created by extending the original region of the input space by 25% and 50 arbitrary sets of patterns are created in the extrapolation region. The performance of the proposed neural models is summarized for these 50 patterns in Figure 4 which shows that the accuracy of the KBNN models is depreciating much slower than that of MLP neural model in the extrapolation region. It may be due to the built-in prior knowledge in the KBNN models that give more information to the patterns not seen during the training.

**Figure 5:**
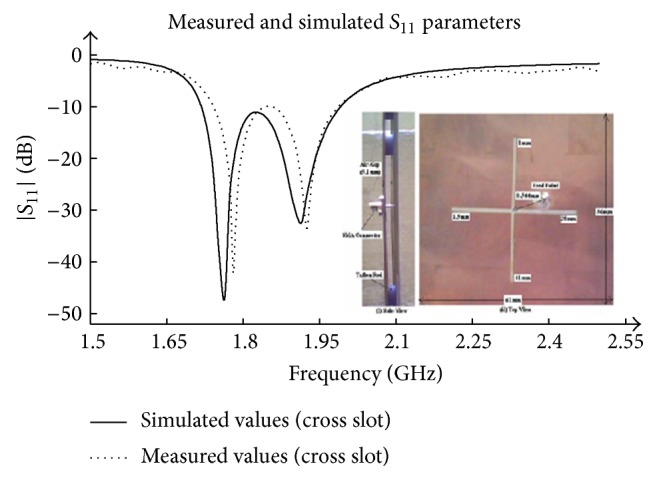
Measured and simulated* S*-parameters.

**Figure 6:**
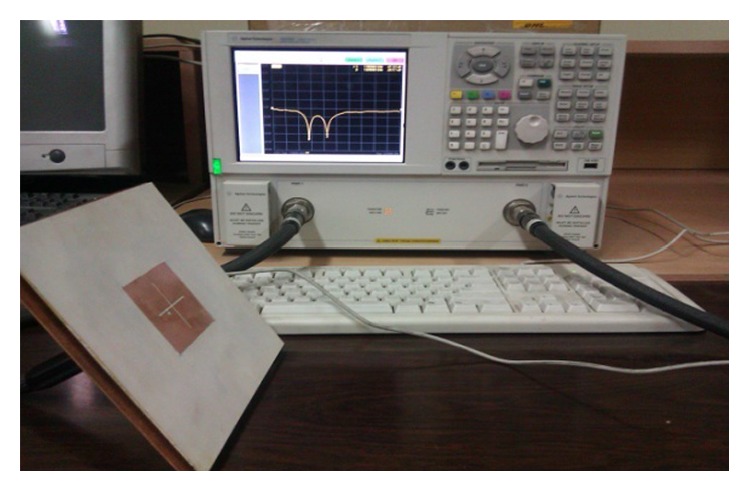
Screenshot of experimental setup.

For validating the work, a prototype is fabricated using RT-Duroid substrate. The patch of dimensions 61 × 56 mm^2^ is etched on the upper side of the substrate whereas an air-gap of 5.1 mm between the substrate and the ground plane is inserted using Teflon rods (Figure 5). The prototype is excited by probe feed, which guides the electromagnetic waves to the feed point. An SMA connector with a 6.8 mm long pin (1 mm for ground plane, 5.1 mm for air-gap, and 0.762 mm for substrate) is used for RF connection. The input reflection coefficients denoted by *S*_11_ of the fabricated prototype are measured using Agilent N5230A network analyzer. A comparison between measured and simulated reflection coefficients is depicted in Figure 4, which shows a good convergence between the two. It is also confirmed here that the Teflon rods do not affect the antenna performance. The computed results are compared with the measured results as mentioned in Table 6 and shown in Figure 5. Thus, a good agreement is achieved between predicted, simulated, and measured dual resonance. The screenshot of experimental setup used during measurement is also shown in Figure 6.

## 6. Conclusion

In this paper, MLP neural networks modeling scheme has been suggested for instantly predicting the slot shape (cross slot, square slot, longitudinal slot, and transverse slot) and slot size (dimensions of predicted slot), simultaneously. This prediction has been carried out for achieving the desired values of dual resonance (in between 1.5 GHz and 3.0 GHz), dual-frequency gains (in between 6.2 dBi and 9.6 dBi), dual-frequency directivities (in between 6.6 dBi and 9.9 dBi), dual-frequency antenna efficiencies (in between 83% and 100%), and dual-frequency radiation efficiencies (in between 85% and 100%). Such a neural approach has been rarely attempted earlier in the open literature. For training of MLP model, 70% of total generated patterns have been used and the performance of the trained model has been tested with the remaining 30% of patterns.

The reliability of MLP neural model is directly linked with adequate number of used training patterns and these patterns have been created using IE3D simulation. The simulation approach has been observed to be a time consuming process because it has been performed for several combinations of each input slot dimension. To reduce the number of training patterns, two knowledge-based schemes based on incorporating prior knowledge in MLP model have also been used. The prior knowledge has been obtained by another trained neural model, known as coarse neural model. The knowledge-based neural approach is very much advantageous when the generation of training patterns is expensive and time consuming as in the case of electromagnetic problems. Both the knowledge-based methods have shown better accuracy even with the least training patterns over MLP neural model with adequate number of training patterns. Further, the difference method has produced more accurate results than that of the PKI method for the proposed problem. For validating the work, predicted results have also been compared with their measured counterparts and found a very good agreement.

## Figures and Tables

**Table 1:** Optimized structural configuration.

Knowledge-based neural method	Structural configuration		
	*m*	*n*	*P*
Difference method **(Coarse model)**	10	8	5
Difference method **(Fine model)**	15	14	5
PKI method **(Coarse model)**	10	10	5
PKI method **(Fine model)**	20	14	5

**Table 2:** Comparison of error versus training algorithm.

Training algorithm [36–39]	Iteration required	Training time (sec.)	Average absolute error in predicting slot size (cm)			
			*x*_1_	*y*_1_	*x*_2_	*y*_2_
BFG	61534	51144	8.445	9.342	7.996	6.998
BR	51975	71539	7.665	9.459	4.995	6.128
SCG	63245	71493	8.110	7.129	8.234	9.665
CGP	54318	41968	9.665	5.776	7.432	6.563
CGF	48953	41132	7.993	7.885	6.997	9.134
OSS	37903	51345	6.772	3.664	4.776	5.771
**LM**	**14562**	**1589**	**0.189**	**0.132**	**0.147**	**0.162**

**Table 3:** Structures comparison of neural models.

Neural model	Model size	Weights	Biases
Standard MLP model	*m* = 10, *n* = 73, *p* = 76, and *q* = 5	6658	154
Difference method	*m* = 10, *n* = 8, and *p* = 5 and *m* = 15, *n* = 14, and *p* = 5	400	32
PKI method	*m* = 10, *n* = 10, and *p* = 5 and *m* = 20, *n* = 14, and *p* = 5	500	34

**Table 4:** Accuracy comparison.

Number of patterns	Neural model	Error in testing
Training = 70% and testing = 30%	MLP method	3.14%
	Difference method	0.57%
	PKI method	1.15%

Training = 50% and testing = 50%	MLP method	14.86%
	Difference method	1.17%
	PKI method	1.49%

Training = 40% and testing = 60%	MLP method	23.81%
	Difference method	1.53%
	PKI method	2.23%

**Table 5:** Performance comparison.

Simulated slot size (mm) and dual resonance (GHz)		Predicted slot size (mm) and dual resonance (GHz)	
Simulated slot size (mm)	Dual resonance (GHz)	Predicted slot size (mm)	Dual resonance (GHz)
*x*_1_ = 38.0000	1.7708 and 1.9221	*x*_1_ = 38.1042	1.7807 and 1.9221
*y*_1_ = 1.5000		*y*_1_ = 1.5013	
*x*_2_ = 1.0000		*x*_2_ = 0.9801	
*y*_2_ = 41.0000		*y*_2_ = 41.1033	

**Table 6:** Comparison of dual resonance.

Dual resonance (GHz)		
Predicted values	Simulated values	Measured values
1.7807 and 1.9176	1.7708 and 1.9221	1.7800 and 1.9250
